# Relationship of cognitive decline with glucocerebrosidase activity and amyloid‐beta 42 in DLB and PD

**DOI:** 10.1002/acn3.52295

**Published:** 2025-03-06

**Authors:** Maria Camila Gonzalez, Linn Oftedal, Johannes Lange, Diego Alejandro Tovar‐Rios, Ole‐Bjørn Tysnes, Claire Paquet, Marta Marquié, Mercè Boada, Daniel Alcolea, Konrad Rejdak, Ewa Papuc, Jakub Hort, Cristian Falup‐Pecurariu, Dag Aarsland, Guido Alves, Jodi Maple‐Grødem

**Affiliations:** ^1^ Department of Quality and Health Technology, Faculty of Health Sciences University of Stavanger Stavanger Norway; ^2^ Centre for Movement Disorders Stavanger University Hospital Stavanger Norway; ^3^ Centre for Age‐Related Medicine Stavanger University Hospital Stavanger Norway; ^4^ Department of Chemistry, Bioscience and Environmental Engineering University of Stavanger Stavanger Norway; ^5^ Department of Neurology Haukeland University Hospital Bergen Norway; ^6^ Neurology Center, Assistance Publique Hôpitaux de Paris, Lariboisière Fernand‐Widal Hospital, INSERMU1144 Université de Paris Paris France; ^7^ Ace Alzheimer Center Barcelona–Universitat Internacional de Catalunya Barcelona Spain; ^8^ CIBERNED, Center for Networked Biomedical Research On Neurodegenerative Diseases National Institute of Health Carlos III Madrid Spain; ^9^ Sant Pau Memory Unit, Department of Neurology, IIB Sant Pau—Hospital de Sant Pau Universitat Autònoma de Barcelona Barcelona Spain; ^10^ Department of Neurology Medical University of Lublin Lublin Poland; ^11^ Department of Neurology, Memory Clinic Charles University, Second Faculty of Medicine and Motol University Hospital Prague Czech Republic; ^12^ Department of Neurology, County Clinic Hospital, Faculty of Medicine Transilvania University Brasov Romania; ^13^ Department of Psychological Medicine Institute of Psychiatry Psychology & Neuroscience King's College London London UK; ^14^ Departement of Neurology Stavanger University Hospital Stavanger Norway

## Abstract

**Objective:**

Dementia with Lewy bodies (DLB) and Parkinson's disease (PD) share clinical, pathological, and genetic risk factors, including *GBA1* and *APOEε4* mutations. Biomarkers associated with the pathways of these mutations, such as glucocerebrosidase enzyme (GCase) activity and amyloid‐beta 42 (Aβ42) levels, may hold potential as predictive indicators, providing valuable insights into the likelihood of cognitive decline within these diagnoses. Our objective was to determine their association with cognitive decline in DLB and PD.

**Methods:**

A total of 121 DLB patients from the European‐DLB Consortium and 117 PD patients from the Norwegian ParkWest Study were included in this study. The four most commonly associated variants of *GBA1* mutations (E326K, T369M, N370S, L444P), *APOEε4* status, and cerebrospinal fluid (CSF) Aβ42 levels and GCase activity were assessed, as well as global cognition using the Mini‐Mental State Examination. Linear mixed‐effects regression models were used to evaluate the association of CSF biomarkers with cognitive decline in each diagnostic group, adjusted for age, sex, education, and genetic mutation profile.

**Results:**

Low CSF Aβ42 levels were associated with accelerated cognitive decline in DLB, whereas reduced CSF GCase activity predicted faster cognitive decline in PD. These associations were independent of *GBA1* gene mutations or *APOEε4* status.

**Interpretation:**

Our study provides important evidence on the relationship between brain Aβ deposition and GCase activity in the Lewy body disease spectrum independent of their genetic mutation profile. This information could be relevant for designing future clinical trials targeting these pathways.

## Background

Dementia with Lewy bodies (DLB) and Parkinson's disease (PD) are neurodegenerative disorders classified as neuronal synuclein diseases. They share various clinical, genetic, and pathological features. In both conditions, abnormal alpha‐synuclein aggregates accumulate in the brain,^1^ which is considered their neuropathological hallmark. Both disorders present a range of symptoms, including motor deficits, neuropsychiatric manifestations, and autonomic dysfunction. Additionally, both disorders exhibit progressive cognitive decline with similar rates of deterioration in the advanced stages of the diseases.^2^ In recent decades, advancements in genetic studies have contributed to breakthroughs in understanding different pathways involved in the development and progression of DLB and PD. Two extensively researched genes associated with DLB and PD are *GBA1* and *APOE*. *GBA* has been identified as a genetic risk factor for the development of DLB and PD. Moreover, besides its role as a genetic risk factor for developing DLB, *APOE* has been shown to influence the presentation and progression of both PD and DLB.^3^, ^4^

The *APOEε4* allele has been found to be associated with impaired Aβ clearance in the brain, leading to the accumulation of amyloid‐beta (Aβ) plaques.^5^ While *APOEε4* and Aβ plaques have been broadly described in both DLB and PD, a major distinction between the two lies in the extent and severity of co‐existing amyloid pathology. DLB is more frequently associated with the presence of Aβ plaques than PD.^6^, ^7^, ^8^ In addition, Aβ biomarkers are more strongly associated with cognitive impairment in DLB than PD, although results show considerable variation dependent on disease stage, follow‐up period, and cognitive measures used.^9^, ^10^, ^11^, ^12^

Glucocerebrosidase (GCase), encoded by the *GBA1* gene, is a critical enzyme involved in the breakdown of glucocerebroside within lysosomes and is implicated in the formation of Lewy bodies in DLB and PD.^13^, ^14^, ^15^ We have recently shown that GCase activity is associated with cognitive decline in PD and holds potential for predicting dementia at the early stages of the disease.^16^, ^17^ However, reports on GCase activity and cognitive decline in DLB are scarce and few studies have studied the connection between GCase activity and cognitive decline across the LBD spectrum.

Contrary to *GBA1/APOEε4* gene carrier status, which remains unchanged over time, GCase activity and Aβ42 levels provide dynamic measures of the related pathways. Understanding the relationship between these biomarkers and cognitive decline is crucial, given the growing interest in disease‐modifying therapies targeting GCase activity and Aβ deposition,^18^, ^19^ in addition to the need for more efficient biomarker‐driven clinical trial designs.

Against this background, we compared the association of cerebrospinal fluid (CSF) GCase activity and Aβ42 levels with longitudinal cognitive decline in two large DLB and PD cohorts from the European‐DLB (E‐DLB) Consortium and the Norwegian ParkWest Study.

## Methods

### Participants

A total of 11 centers recruited patients with DLB (*n* = 6) or PD (*n* = 5). Eligible cases were required to have undergone CSF sampling and have a minimum set of clinical and demographic information. Probable DLB^20^ (*n* = 134) subjects were mostly referrals from outpatient clinics, including memory, movement disorder, geriatric medicine, psychiatric, and neurology clinics with cross‐center harmonization of diagnostic procedures from the E‐DLB consortium.^21^ Newly diagnosed PD^22^ cases (*n* = 120) were identified from the Norwegian ParkWest study, a prospective population‐based longitudinal cohort study of patients with incident PD.^23^ The local ethics committees approved each study and all participants signed written informed consent. We harmonized demographic and medical history data both at baseline and during the follow‐up period. To assess global cognition, we utilized the Mini‐Mental State Examination (MMSE).^24^ Motor severity was evaluated using the Unified PD Rating Scale (UPDRS) part III or the Movement Disorder Society‐UPDRS (MDS‐UPDRS) part III.^25^ For a subset of patients we used the simplified conversion method to transform UPDRS scores to MDS‐UPDRS part III.^26^ Additional information on the cohorts can be found in Table S1.

### CSF biomarkers

A detailed overview of CSF sample collection, handling, and storage, and reference CSF Aβ42 cutoff values are available in Table S1.

GCase activity was measured in both DLB and PD patients with the same validated fluorometric in vitro assay.^27^ Detailed information on the methods used for GCase activity and CSF total protein content measurement in PD, including assay procedures, quality control measures, has been previously published,^16^ this included the 117 patients with PD included in this work. Briefly, CSF samples were diluted 1:2 in assay buffer and supplemented with 4‐methylumbelliferyl‐β‐D‐glucopyranoside as a substrate. After incubation at 37°C for 3 h, the reaction was stopped by adding 0.2 M glycine pH 10.2 and the concentration of the fluorescent cleavage product, 4‐methylumbelliferyl, was measured (Excitation: 360 nm/Emission: 446 nm). All samples were analyzed in triplicates. Single replicate values deviating more than twofold from the mean of the other two replicates were identified as outliers and excluded (*n* = 12; 6.9%). Since PD and DLB samples were analyzed on three separate dates, each run included quality control samples on every plate to ensure reliability and consistency, demonstrating satisfactory interassay reproducibility. The lower limit of quantification for GCase activity in the DLB cohort assay was determined to be 0.190 mU/mL, corresponding to a signal 10 standard deviations above background. Additionally, the lower limit of detection, defined as a signal 2 standard deviations above background, was found to be 0.053 mU/mL. Mean GCase activity in the DLB CSF samples was 0.97 mU/mL (range 0.1–1.9). Mean sample CV% was 7.4 (range 0.3–30.0). Only two samples exceeded a CV of 20%. Intraassay CV% was 4.7 (five plates, quality control samples on each plate). One unit of GCase activity was defined as amount of enzyme that hydrolyses 1 nmol of substrate/min at 37°C. Total protein content was measured using a bicinchoninic acid assay (Thermo Fisher Scientific). Samples were run in duplicate at 1:2 dilution. The LLOQ was 0.14 mg/mL. Mean protein content was 0.8 mg/mL (range 0.5–1.5). Mean sample CV% was 3.3 (range 0.04–27.7). Only two samples exceeded a CV of 20%. Intraassay CV% was 9.5%. One sample was excluded due to technical reasons (protein concentration above the ULOQ of 4.2 mg/mL). The final DLB cohort consisted of 121 patients.

There was a positive correlation between the total protein concentration and the GCase activity per mL of CSF in both groups (DLB: Kendall tau = 0.112; *P* = 0.01; PD group: Kendall tau *b* = 0.268, *P* < 0.001). To facilitate further analysis, the GCase enzymatic activity was adjusted by normalizing it to the CSF total protein content, resulting in specific activity reported as mU/mg.^28^

### Genotyping

We obtained data on the four most commonly associated variants of *GBA1* mutations (E326K, T369M, N370S, L444P) with DLB and PD risk, along with information on the apolipoprotein E (*APOE*) ε4 allele on a subset of participants (Table S2). The procedures for genotyping PD patients have been described previously.^3^ For DLB patients, information on *GBA1* and *APOE* status was available for one cohort from a previous whole exome sequencing effort.^29^ For the remaining DLB cohorts, data on the genetic status was acquired using TaqMan genotyping assays (assay ID C___3084793_20/rs7412; C____904973_10/rs429358; C__57592026_10/rs2230288; C__64675913_10/rs75548401; Custom assay/ rs76763715; ThermoFisher) and the StepOnePlus Real‐Time PCR System (Applied Biosystems). Finally, to identify mutations in the L444P variant, we employed a polymerase chain reaction–restriction fragment length polymorphism (PCR‐RFLP) technique. The PCR‐RFLP assay involved amplifying a fragment containing exons 8–11 of the *GBA1* gene using the primers 5′‐TGTGTGCAAGGTCCAGGATCAG‐3′ and 5′‐ACCACCTAGAGGGGAAAGTG‐3′ with the MyTaq™ Mix polymerase from Meridian Bioscience. Subsequently, the PCR product was subjected to digestion using the *Nci*I restriction enzyme. For analysis, patients were grouped by genotype: *APOE*, carriers of the ε4 allele versus non‐carriers; *GBA1*, carriers of one or more non‐synonymous *GBA1* mutations versus non‐carriers.

### Statistical analysis

Descriptive statistics were performed with means and standard deviations computed for continuous variables and frequencies and percentages for categorical variables. Group differences were compared using *t*‐tests and *χ* ^2^ tests, as appropriate. MMSE scores were transformed using the logarithm of (31‐MMSE), to reduce skewness.

CSF Aβ42 concentrations were divided into high or low, based on local reference standards (Table S1). Due to differences in frozen storage time between the two diagnostic groups (median date for PD sample collection: October 2005, average frozen storage time: 15.4 years; for DLB: median sample collection date: 30 November 2017, average frozen storage time: 3.5 years), a known preanalytic factor influencing GCase robustness,^30^ we conducted stratified analyses based on diagnoses. GCase activity was devided into tertiles. For the DLB group, the cutoffs were as follows: >1.46 mU/mg (high activity), 1.46–0.97 mU/mg (intermediate activity), and <0.97 mU/mg (low activity). For the PD group, the corresponding cut‐offs were 1.12 mU/mg (high activity), 0.80–1.12 mU/mg (intermediate activity) and <0.80 mU/mg (low activity).^16^

To assess the association between biomarker levels and cognition, we ran linear mixed‐effects models for each biomarker with MMSE as the dependent variable using all available MMSE scores (the number of patients with DLB and PD diagnosis and their total or last recorded visit as well as total number of annual follow‐up assessments are available on the Fig. S1). For modeling, the low GCase activity group and the high Aβ42 level group were set as the reference, respectively. These models incorporated an interaction term between time and each GCase/Aβ42 group as a fixed effect as well as a random intercept and slope for each participant to capture individual variations in baseline cognition and cognitive decline. A random intercept for each study center was also included. The models were then adjusted for potential confounding variables, including age, sex, years of education and gene mutation status for *GBA1* and *APOE*. In order to explore the relative importance of the different predictors, effect sizes were calculated as standardized coefficients to evaluate the change in the dependent variable in standard deviation units. Hypotheses were rejected in each model on an alpha level of 0.05 (two‐tailed). IBM SPSS Statistics 26 was used for data management, STATA 17 for data manipulation and modeling, and R version 4.0.5 for graphics.

## Results

### Characteristics of the DLB and PD participants at baseline

Baseline characteristics are provided in Table 1. The maximum follow‐up time was 11.7 years (median 10.9, IQR 2.9) for the PD group and 8.1 years (median 2.1, IQR 2.3) for the DLB cohort. Both groups exhibited comparable distributions in terms of sex, MDS‐UPDRS part III, CSF Aβ42 and *GBA1/APOEε4* status. As anticipated, DLB patients were older and displayed lower MMSE scores at baseline.

**Table 1:** Cohort overview at baseline.

	DLB	PD	Total	*P* value
Total, *n* (%)	121 (51.0)	117 (49.0)	238 (100.0)	
Age	71.7 ± 7.1	66.7 ± 9.5	69.2 ± 8.7	<0.01
Sex (Male), *n* (%)	66 (54.6)	76 (65.0)	142 (59.7)	0.10
Year of education	9.8 ± 4.5	11.1 ± 3.1	10.4 ± 3.9	<0.01
MMSE	22.7 ± 4.8	27.7 ± 2.4	25.2 ± 4.6	<0.01
MDS‐UPDRS III	29.9 ± 19.4	29.0 ± 12.5	29.2 ± 14.2	0.75
*GBA1, n* (%)				
Non‐carriers	91 (95.8)	103 (88.8)	194 (91.9)	0.06
Carriers	4 (4.2)	13 (11.2)	17 (8.1)	
*APOEε4, n* (%)				
Non‐carriers	46 (61.3)	81 (69.2)	127 (66.2)	0.26
Carriers	29 (38.7)	36 (30.8)	65 (33.9)	
CSF Aβ42, *n* (%)				
High	53 (45.7)	45 (42.5)	98 (44.1)	0.63
Low	63 (54.3)	61 (57.6)	124 (55.9)	
GCase, mU/mg	1.20 (0.5)	1.0 (0.4)	‐	‐
CSF total protein, μg/mL	83.0 (16.3)	85.4 (19.6)	‐	‐

Values are presented as mean ± SD, unless stated.

CSF, cerebrospinal fluid; MMSE, Minimal‐ Mental State Examinations.

### Impact of GCase activity on cognition in DLB and PD

In our initial linear mixed‐effects model, we explored the influence of baseline GCase activity on global cognitive function in DLB and PD throughout the follow‐up period.

**Figure 1:**
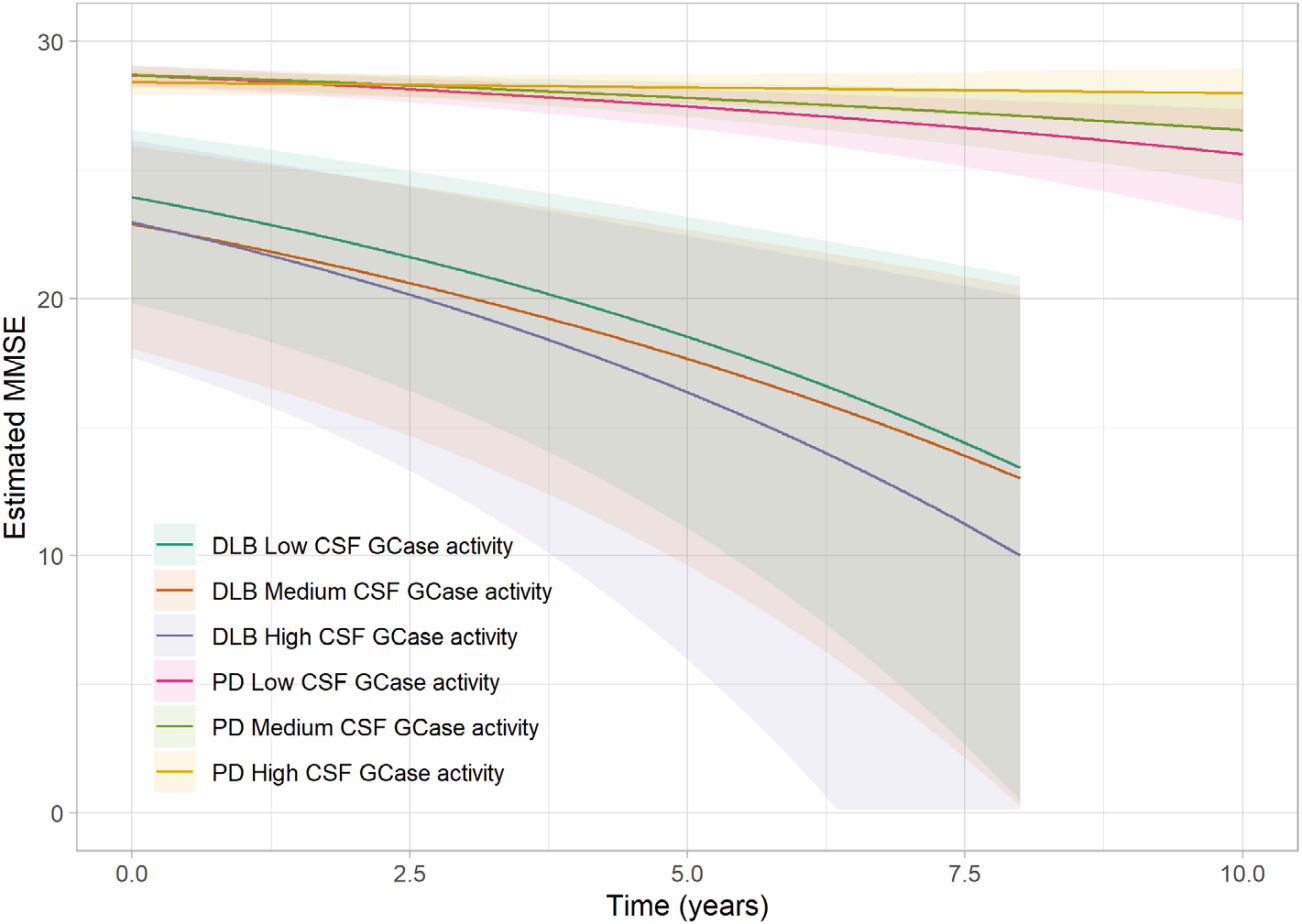
Estimated impact of GCase activity on cognition in DLB and PD.

Cross‐sectional comparisons at baseline demonstrated no significant differences in MMSE scores between the three GCase activity groups in either diagnostic group. Similarly, longitudinal analyses revealed no differences in rate of cognitive decline during follow‐up between DLB patients with low, intermediate or high GCase activity. In contrast and as previously shown by our group,^17^ individuals with PD with low GCase activity experienced more rapid cognitive decline, with a decline rate of −0.34 MMSE points per year (95% CI −0.62; −0.17, *P* = 0.02) compared to those with high GCase activity who experienced a decline of −0.04 MMSE points per year (95% CI −0.14; 0.02).^17^ Importantly, our findings reveal that the observed rates of cognitive decline remained unaffected by the presence of *GBA1* and *APOEε4* carrier status (Table 2, Fig. 1). Moreover, the effect size for low GCase activity on cognition in the PD group was found to be 0.23, while for the DLB group, the effect size was 0.01.

**Table 2:** Association of GCase activity with annual changes in Mini‐Mental State Examination (MMSE) as determined by linear mixed models.

MMSE	Unadjusted			Adjusted		
	Est.	Std. Err.	*P*‐value	Est.	Std. Err.	*P*‐value
*DLB*						
GCase lower tertile (ref)						
Intercept	1.99	0.17	<0.01	0.59	0.84	‐
Time	0.10	0.02	<0.01	0.11	0.03	‐
GCase middle tertile (vs. ref)						
Intercept	0.03	0.15	0.84	0.14	0.19	0.47
Time	−0.01	0.03	0.86	−0.01	0.04	0.71
GCase higher tertile (vs. ref)						
Intercept	0.22	0.16	0.19	0.13	0.22	0.55
Time	0.02	0.04	0.66	0.01	0.05	0.90
GBA1 status	‐	‐	‐	−0.04	0.37	0.91
APOEε4 status	‐	‐	‐	0.49	0.13	<0.01
Age	‐	‐	‐	0.02	0.01	0.04
Sex (female vs. male)	‐	‐	‐	0.14	0.13	0.29
Years of education	‐	‐	‐	−0.04	0.02	0.03
*PD*						
GCase lower tertile (ref)						
Intercept	0.94	0.09	<0.01	−0.39	0.47	‐
Time	0.08	0.02	<0.01	0.08	0.02	‐
GCase middle tertile (vs. ref)						
Intercept	−0.06	0.13	0.65	−0.01	0.12	0.96
Time	−0.01	0.03	0.67	−0.02	0.03	0.55
GCase higher tertile (vs. ref)						
Intercept	−0.10	0.13	0.43	0.11	0.13	0.38
Time	−0.07	0.03	0.03	−0.07	0.03	0.02
GBA1 status	‐	‐	‐	0.12	0.14	0.42
APOEε4 status	‐	‐	‐	0.04	0.10	0.71
Age	‐	‐	‐	0.03	0.01	<0.01
Sex (female vs. male)	‐	‐	‐	−0.21	0.10	0.03
Years of education	‐	‐	‐	−0.04	0.02	0.01

### Impact of Aβ42 status on cognition in MMSE in DLB and PD

**Figure 2:**
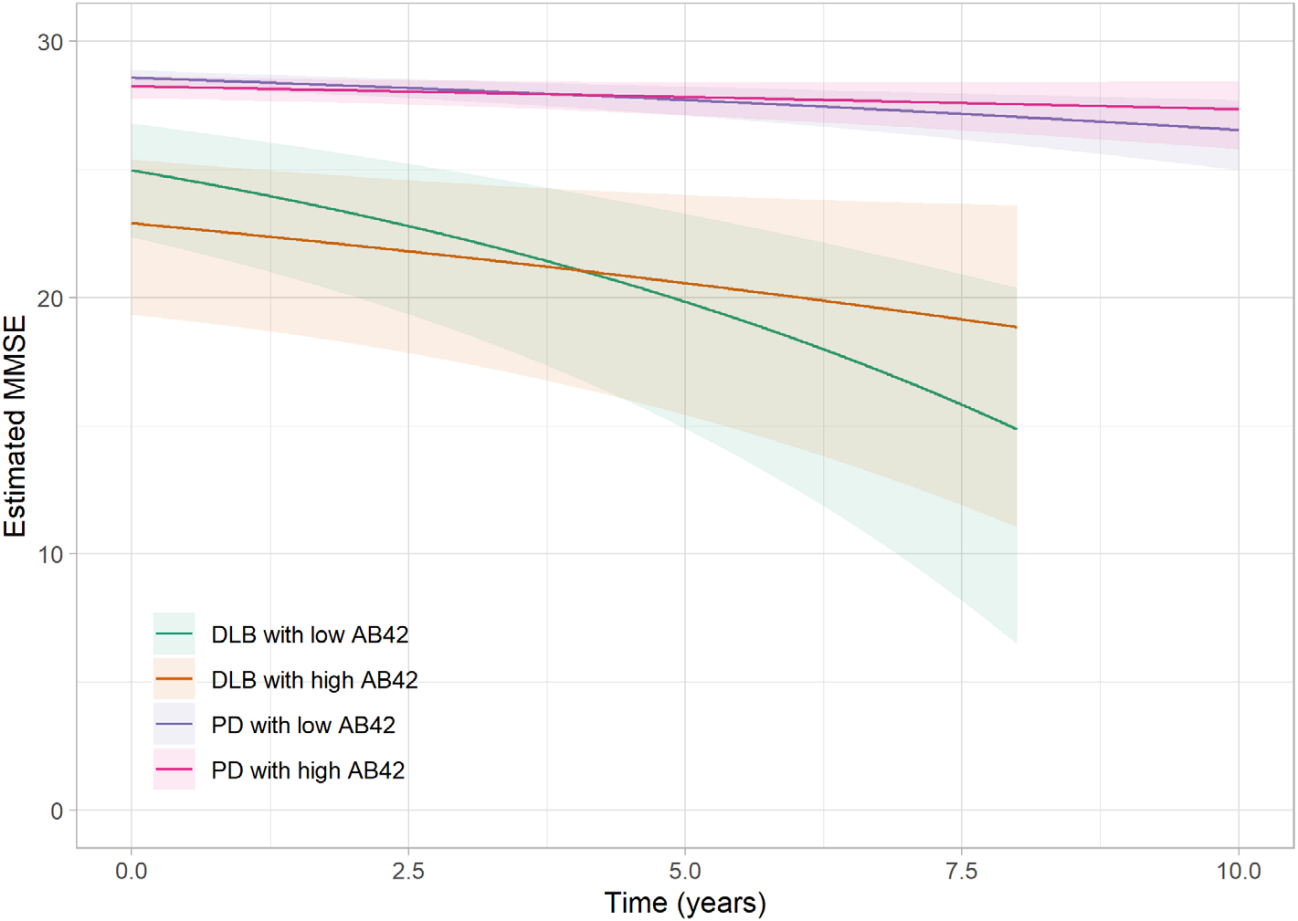
Estimated impact of Aβ42 status on cognition in MMSE in DLB and PD.

We further examined the influence of CSF Aβ42 levels on global cognitive function in DLB and PD throughout the follow‐up period. At baseline, cross‐sectional analyses showed that DLB patients with low versus high Aβ42 CSF levels did not differ in their cognitive performance in the unadjusted (*P* = 0.75) but in the adjusted model (*P* = 0.03). In longitudinal analyses, DLB patients with low baseline Aβ42 CSF levels experienced a more rapid cognitive decline (−1.26 MMSE points per year, 95% CI −1.98 to −0.80, *P* = 0.03) during follow‐up compared to DLB patients with high baseline Aβ42 CSF levels (−0.51 MMSE points per year, 95% CI −1.03 to −0.22). These results remained unaffected by the presence of *GBA1* gene mutations, *APOEε4* status, and demographic factors (Table 3, Fig. 2). A similar trend was observed in the PD group, where the predicted decline for patients with low baseline Aβ42 CSF levels was more than doubled compared to those with high Aβ42 CSF levels (−0.22 MMSE points per year vs. −0.09 MMSE points per year). However, this difference did not reach statistical significance (*P* = 0.06). Moreover, the effect size for low CSF Aβ42 on cognition in the PD group was found to be 0.11, while for the DLB group, the effect size was 0.18.

**Table 3:** Association of Aβ42 status with annual changes in Mini‐Mental State Examination (MMSE) as determined by linear mixed models.

MMSE	Unadjusted			Adjusted		
	Est.	Std. Err.	*P*‐value	Est.	Std. Err.	*P*‐value
*DLB*						
Aβ42 high (ref)						
Intercept	1.98	0.13	<0.01	0.92	0.77	‐
Time	0.06	0.02	<0.01	0.05	0.03	‐
Aβ42 low (vs ref)						
Intercept	0.04	0.11	0.75	−0.29	0.14	0.03
Time	0.05	0.02	0.03	0.07	0.03	0.03
APOEε4 status	‐	‐	‐	0.55	0.13	<0.01
GBA1 status	‐	‐	‐	−0.06	0.32	0.86
Age	‐	‐	‐	0.02	0.01	0.07
Sex (female vs male)	‐	‐	‐	0.22	0.13	0.09
Years of education	‐	‐	‐	−0.03	0.02	0.05
*PD*						
Aβ42 high (ref)						
Intercept	0.96	0.08	<0.01	−0.21	0.50	‐
Time	0.03	0.02	0.06	0.03	0.02	‐
Aβ42 low (vs. ref)						
Intercept	−0.09	0.10	0.41	−0.13	0.11	0.23
Time	0.04	0.02	0.08	0.03	0.02	0.19
APOEε4 status	‐	‐	‐	0.00	0.10	0.96
GBA1 status	‐	‐	‐	0.22	0.17	0.19
Age	‐	‐	‐	0.02	0.01	<0.01
Sex (female vs. male)	‐	‐	‐	−0.22	0.11	0.04
Years of education	‐	‐	‐	−0.03	0.02	0.11

## Discussion

In this longitudinal study, we investigated the relationship of CSF GCase activity and Aβ42 levels with cognitive decline across the LBD spectrum using two multicenter cohorts with DLB and PD. We found that CSF Aβ42 levels predicted cognitive decline in DLB, whereas GCase activity was more closely related to cognitive decline in PD. For both, the observed associations were independent of *GBA1* or *APOEε4* carrier status. These findings provide insight into the relative importance of the respective pathways in disease progression and have implications for trial design for these disease groups.

Both *GBA1* variants and further GCase dysfunction appear to predispose to accelerated α‐synuclein aggregation and Lewy body pathology.^13^, ^31^ While many studies have shown an association between *GBA1* mutations and cognitive progression in PD and DLB,^32^, ^33^ there has been limited research directly comparing the relationship of GCase activity and cognitive progression across the PD spectrum,^17^, ^28^, ^34^, ^35^ and to the best of our knowledge, none in DLB. We have previously and here shown that shown that lower GCase activity is predictive of the annual rate of decline in MMSE score in PD.^16^, ^17^ However, this was not the case in DLB. This is striking given that *GBA1* mutations are more prevalent in DLB compared to PD, and postmortem studies have shown that GCase activity is reduced in patients with DLB with and without *GBA1* muations.^32^ Although the reasons for this discrepancy are unclear, there are a number of potential explanations. While *GBA1* mutations are typically associated with reduced GCase activity,^36^ impairments in GCase function can manifest through mechanisms beyond genetic mutations.^13^ In newly diagnosed PD, characterized by alpha‐synuclein pathology as a central feature, GCase dysfunction may predominantly contribute to cognitive decline due to its direct involvement in alpha‐synuclein metabolism.^37^, ^38^, ^39^ However, in DLB, cognitive impairment may arise from a complex interplay of multiple pathological processes,^40^ and amyloid pathology may overshadow the predictive value of GCase activity and be more influential in driving cognitive decline in this disease. Additionally, factors such as neural reserve and cognitive abilities,^41^ which are generally more preserved in newly diagnosed PD cases compared to DLB,^42^ may influence the lack of association observed. Furthermore, the small effect size in our study suggests that larger cohort sizes or extended follow‐up periods may be necessary to observe significant associations. However, the E‐DLB cohort is characteristic of the participants available for clinical trials, and the median length of follow‐up aligns with typical trial settings. Thus, our findings imply that GCase activity may not be directly applicable to clinical trials mirroring our DLB population.

CSF Aβ42 on the other hand, is an established biomarker of cerebral Aβ deposition.^43^ In advanced stages, nearly half of DLB patients exhibit abnormal levels indicative of Aβ co‐pathology.^44^ In our study DLB cases with low CSF Aβ42 values experienced steeper decline in MMSE score than those with high Aβ42 profiles, independent of *APOEε4* status. These findings highlight a consistent association between low CSF Aβ42 levels and a more aggressive disease course in DLB.^45^ By contrast, evidence regarding of Aβ brain deposition markers and cognitive decline in PD varies significantly based on a number of factors, including disease stage of PD and outcome measures.^9^, ^10^, ^11^, ^46^ We observed that low Aβ42 CSF levels showed a trend toward faster MMSE decline in the PD group, although the statistical significance was marginal. In contrast, a previous study of our ParkWest cohort demonstrated an increased risk of dementia development within 5 years among those with low CSF Aβ42 at diagnosis of PD.^10^ Combined, these findings are aligned with clinical‐pathological studies suggesting that Aβ brain deposition is most prominent in PD patients with earliest cognitive decline, while Lewy body pathology is thought to be responsible for cognitive decline in later stages of the disease.^47^ This emphasizes the need for a more nuanced understanding of the disease for accurate predictions and targeted interventions.

### Strenghts and limitations

Our study has strengths and limitations. Strengths include the international multicenter design to recruit patients, the relatively large sample size with available CSF, the standardized clinical assessments, and the longitudinal follow up. Our study also has limitations. First, we recognize the differences in recruitment and follow‐up between the population‐based PD cohort and the clinical‐based DLB cohorts in this study. Achieving population representativeness in DLB is challenging, and the limited length of follow‐up in this patient group is often due to the aggressive disease course and high mortality. Second, both DLB and PD are frequently misdiagnosed and we only have postmortem confirmation for a subgroup of patients (*n* = 42)^48^ from the ParkWest cohort. However, diagnoses for both groups were established by specialists over continued follow up and met widely acknowledged diagnostic criteria at final visit. Third, we assessed cognitive decline using the MMSE rather than detailed neuropsychological testing, which is more sensitive to detect subtle cognitive changes, particularly in executive or visuospatial functioning that are commonly affected in DLB and PD patients. Still, our findings hold significance as the MMSE is the most widely adopted cognitive screening tool and is included as the primary outcome measure in several ongoing clinical trials.^18^ Given the retrospective design of our study and the variability in biomarker collection across cohorts (specially for the DLB cohort), there were instances of missing data, particularly in genetic markers and other clinical considerations such as motor symptoms. This limitation is most evident in the analysis of the 4 *GBA* mutations variants, where incomplete data led to small sample sizes. As a result, we were unable to effectively explore the effects of *GBA* mutations categorized by severity. With respect to the biomarkers in this study, CSF Aβ42 levels in DLB patients were assessed locally at each center using different immunoassays, which could introduce variability. To account for this we harmonized the data using respective center‐specific reference values and the results were robust to possible variation introduced by the specific assay employed. Another consideration is the long storage time of the PD samples for the GCase assays, which introduced limitations in conducting direct comparisons between the two diagnostic groups. Finally, the method we chose for assessing GCase activity has some inherent limitations, such as not accounting for endogenous factors that could influence GCase activity, as well as the unknown correlation between CSF and tissue activity when measured simultaneously. However, a key strength is that our selection of the GCase measurement assay is one of the most widely adopted methods for assessing GCase activity in patients' biofluids, providing excellent sensitivity and performed consistently at a single site.

## Conclusions

Our study provides important evidence on the relationship between brain Aβ deposition and GCase activity in the Lewy body disease spectrum and its association with cognitive decline. These findings offer valuable insights into the shared pathological pathways of both DLB and PD, enhancing our understanding of their expected progression independent of their genetic status. These results hold particular relevance for shaping future clinical trials focused on therapeutic interventions targeting these pathways. Additional studies, including the prodromal stages of these disorders, would be valuable for the field.

## Author Contributions

M.C.G.: Conception of work, methods, performed the analysis, writing—reviewing, and editing. L.O: Methods, writing‐ reviewing, and editing. J.L: Methods, writing—reviewing, and editing. D.A.T‐R: Processed the data, performed the analysis, and designed the figures. O‐B.T, C.P, M.M, M.B, D.Al, K.R, E.P, J.H, C.F‐P: Contributed to clinical data collection and sample preparation, writing—reviewing, and editing. D.A, G.A, J.M‐G: Methods, contributed to clinical data collection and sample preparation, writing—reviewing, and editing. All authors discussed the results and contributed to the final manuscript.

## Funding Information

The PARKWEST study was supported by the Research Council of Norway (grant# 177966), the Western Norway Regional Health Authority (grant# 911218 and # 911949), and Reberg legacy and the Norwegian Parkinson's Research Foundation. The University of Stavanger supported MCG. The Czech Brain Aging Study (www.cbas.cz) was supported by project number LX22NPO5107 (MEYS): Financed by European Union—Next Generation EU. Sant Pau: This work was supported by research grants from Institute of Health Carlos III (ISCIII), Spain PI18/00435, PI22/00611, INT19/00016, INT23/00048 to D.Al., and by the Department of Health Generalitat de Catalunya PERIS program SLT006/17/125 to D.Al.

## Conflict of Interest

The authors declare no competing financial interest.

## Supporting information


Data S1.

## Data Availability

Qualified external researchers can request access to anonymized patient‐level data, respecting patient informed consent, from the corresponding author on reasonable request.
