# Supplementary material for: Relationship of cognitive decline with glucocerebrosidase activity and amyloid‐beta 42 in DLB and PD
Source: Ann Clin Transl Neurol. 2025 Mar 6;12(5):915–24. doi: 10.1002/acn3.52295 (PMC12093344; doi:10.1002/acn3.52295)
Supplement: Supplementary file 1 — Data S1. [file ACN3-12-915-s001.docx]

**Supplementary Table 1.** Overview of CSF sample collection, handling, storage, freeze thaw cycles and procedures

| **Center** | **N (%)** | **Maximum years of follow-up** | **Collection** | **Centrifuging** | **Storage** | **AD-CSF Analysis Essay** | **Cut off values [pg/mL]** | **Number of freeze–thaw events *** |
| --- | --- | --- | --- | --- | --- | --- | --- | --- |
| ParkWest- Southwestern Norway | PD  117 (49.4%) | 11.7 | Polypropylene tube | Centrifuged at 2000g for 10 minutes at 4°C | Stored in polypropylene tubes at -80°C | Human Aβ peptide Ultra-Sensitive Kits (MSD) | Aβ42 < 376^1^ | 1 |
| Université de Paris APHP Laiboisière Cognitive Neurology Center | DLB  42  (17.1%) | 8.1 | Polypropylene tube | Centrifuged at 2000g for 10 minutes at 4°C | Stored in polypropylene tubes at -80°C | INNOTEST Double sandwich ELISAs  Roche Cobas | Aß42 < 730  Aß42 < 860 | 1 |
| County Emergency Clinic Hospital Brasov | DLB  2  (0.8%) | 3.5 | Polypropylene tube | Centrifuged at 2000g for 10 minutes at 4°C | Stored in polypropylene tubes at -80°C | N/A | N/A | 1 |
| Medical University of Lublin | DLB  8  (3.4%) | 2.2 | Polypropylene tube | Centrifuged at 2000g for 10 minutes at room temperature (20°C) | Stored in polypropylene tubes at -80°C | INNOTEST Double sandwich ELISAs | Aβ42 <375 | 1 |
| University Hospital Motol | DLB  13  (5.4%) | 5.7 | Polypropylene tube | Centrifuged at 2000g for 10 minutes at room temperature (20°C) | Stored in polypropylene tubes at -80°C | Lumipulse | Aβ42 < 620 | 1 |
| Ace Alzheimer Center Barcelona, Spain | DLB  46  (19.3%) | 4.7 | Polypropylene tube | Centrifuged at 2000g for 10 minutes at 4°C | Stored in polypropylene tubes at -80°C | Lumipulse | Aβ42 < 796 | 0 |
| Hospital de la Santa Creu i Sant Pau_­_, Spain | DLB  11  (4.6%) | 7.1 | Polypropylene tube | Centrifuged at 2000g for 10 minutes at 4°C | Stored in polypropylene tubes at -80°C | Lumipulse | Aβ42 < 637 | 1 |

*Freeze–thaw events prior to analysis .Abbreviations: (CSF) Cerebrospinal fluid, (AD)-CSF Alzheimer’s Disease Cerebrospinal fluid biomarkers, (DLB) Dementia with Lewy bodies, (PD) Parkinson's Disease

**Supplementary Table 2.**

|  | **DLB** | **PD** | **Total** |
| --- | --- | --- | --- |
| ***APOEε4 n*** | 76 | 117 | 193 |
| *Carrier n (%)* | 29 (38.1) | 36 (30.7) | 65 (33.6) |
| ***GBA n*** | 95 | 116 | 212 |
| *Carrier n (%)* |  |  |  |
| *N370S* | 2 (2.1) | 0 (0.0) | 2 (0.9) |
| *T369M* | 2 (2.1) | 5 (4.3) | 7 (3.3) |
| *E326K* | 1 (1.3) | 8 (6.9) | 9 (4.7) |
| *L444P* | 1 (1.3) | 0 (0.0) | 1 (0.5) |

Supplementary Figure 1. A. Number of dementia with Lewy bodies (DLB) and Parkinson ’s disease (PD) patients completing annual follow-up assessments. B. Number of patients with DLB and PD diagnosis and their total or last recorded visit.


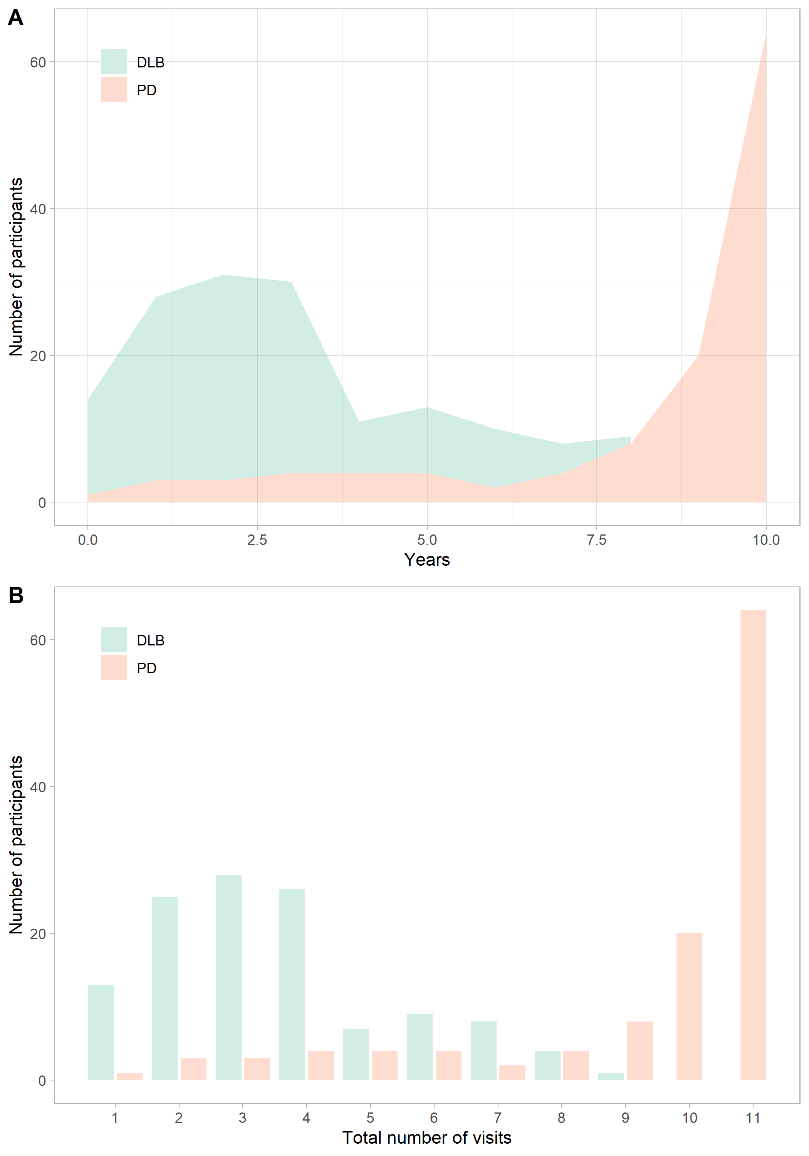


1. Alves G, Lange J, Blennow K, et al. CSF Aβ42 predicts early-onset dementia in Parkinson disease. Neurology 2014;82(20):1784-1790.
